# Effects of acute ethanol intoxication in an ovine peritonitis model

**DOI:** 10.1186/s12871-018-0537-1

**Published:** 2018-06-19

**Authors:** Koji Hosokawa, Fuhong Su, Fabio Silvio Taccone, Emiel Hendrik Post, Jacques Creteur, Jean-Louis Vincent

**Affiliations:** Department of Intensive Care, Erasme University Hospital, Université Libre de Bruxelles, Brussels, Belgium

**Keywords:** Ethanol intoxication, Sepsis, Septic shock, Renal blood flow, Brain perfusion, Lactate/pyruvate

## Abstract

**Background:**

Acute ethanol intoxication has been shown to have contrasting effects on outcomes in sepsis. The aim of this study was to explore the effects of acute ethanol intoxication on hemodynamics, renal function, brain perfusion and lactate/pyruvate in an ovine sepsis model.

**Methods:**

Anesthetized, mechanically ventilated female sheep were randomized to an ethanol group (*n* = 7), which received 1 g/kg ethanol diluted in intravenous (i.v.) saline infusion or a control group (*n* = 7), which received the same volume of i.v. saline. Both groups received the treatment for a period of 2 h prior to induction of sepsis by intraperitoneal injection of feces. Other treatment included fluid resuscitation but no vasopressors or antibiotics. Global hemodynamics, renal blood flow, brain cortex laser Doppler flowmetry and microdialysis analyses were recorded hourly.

**Results:**

In the ethanol group, blood ethanol concentrations were 137 ± 29 mg/dL at the time of feces injection and decreased to become undetectable by 12 h. Arterial hypotension occurred earlier in the ethanol than in the control group (8 [7–12] vs. 14 [11–20] hours, *p* = 0.03). Lactate levels increased to > 2 mmol/L earlier in the ethanol group. Renal dysfunction (9 [6–13] vs. 13 [12–15] hours, *p* = 0.05) and oliguria (urine output < 0.5 mL/kg/h; 10 [7–12] vs. 13 [12, 13] hours, *p* = 0.01) developed earlier in the ethanol than in the control group. Brain blood flow and lactate/pyruvate were unaffected. There was no significant difference in survival time.

**Conclusions:**

Acute ethanol intoxication in this model of peritonitis resulted in earlier development of shock and renal dysfunction but did not alter brain perfusion and metabolism or short-term survival.

**Electronic supplementary material:**

The online version of this article (10.1186/s12871-018-0537-1) contains supplementary material, which is available to authorized users.

## Background

Sepsis and septic shock remain a major concern with high associated morbidity and mortality [1–4]. Chronic ethanol abuse is known to be associated with worse outcomes from sepsis [5–7], but the impact of acute ethanol intoxication is controversial. In animal studies, despite consistent reports of increased mortality, blood pressure increased in one murine endotoxemia model exposed to acute ethanol loading (100 mg/kg/h for 2 h by intravenous infusion) [8] but decreased in a rat endotoxemia model (intra-peritoneal ethanol injection of 2.2–5.5 g/kg) [9]. No specific cardiovascular effects were observed in studies using a pig endotoxemia model with ethanol loading (3 g/kg by gastric lavage) [10]. In an observational study including 11,850 adult patients [11], a detectable blood ethanol concentration at hospitalization was associated with decreased odds of 30-day mortality and a blood ethanol concentration > 160 mg/dL was associated with significantly decreased odds of developing sepsis and bloodstream infection. In another observational study including 31,364 emergency patients with acute ethanol intoxication, the presence of sepsis symptoms, such as tachycardia, hypotension, hypothermia, fever and hypoxia, was associated with increased odds of critical illness [12]. Acute ethanol intoxication has also been reported to be independently associated with an increase in sepsis complications in patients with traumatic brain injury (TBI) [13, 14]. In 23,983 patients with TBI, pneumonia and urinary tract infections were more common in patients with acute ethanol intoxication than in those without [15]. Similar findings were reported in patients with traumatic spinal cord injury [16]. Lower systemic interleukin (IL)-6 levels and leukocyte counts have been reported in TBI patients with acute ethanol intoxication [17], suggesting an immunosuppressive effect of ethanol, although in this study there were no significant differences in occurrence of sepsis or pneumonia in patients with positive or negative ethanol tests.

There are no specific data regarding the effects of acute ethanol intoxication on the occurrence of specific organ dysfunctions in patients with sepsis. Thus, we tested the hypothesis that acute ethanol intoxication may have deleterious hemodynamic effects that could affect kidney and brain perfusion and brain lactate/pyruvate ratio in a large sheep model of fecal peritonitis.

## Methods

The local animal ethics committee at the Université Libre de Bruxelles approved the present study (381 N) and we followed the EU Directive 2010/63/EU for animal experiments and the ARRIVE guidelines for animal research.

Female *Ovis aries* sheep (aged 6–9 months) were obtained from a local farm (BE 400108–48). The sheep were allowed to adapt to the animal facility for 1 week prior to the study and were then fasted overnight. We chose to use sheep of just one sex to reduce heterogeneity and selected female sheep because bladder catheterization is easier. On the day of the study, sheep were randomized to an ethanol group (*n* = 7) that received a 1 g/kg infusion of ethanol (Ethanol 96° Sterop, 96%, diluted to 20% in NaCl 0.9%) and a control group (*n* = 7) that received the same volume of saline. The ethanol/control infusion was continued throughout the surgical preparation until sepsis induction (about 2 h). The dose of ethanol and the duration of administration were chosen to achieve a target concentration of blood ethanol of 100–150 mg/dL to limit any major metabolic and hemodynamic changes during infusion and to achieve elimination of blood ethanol within 10–12 h. This level was chosen based first on results from a retrospective study by Stehman et al. in critically ill patients showing that a detectable blood ethanol level > 80 mg/dL at hospitalization was associated with significantly decreased odds of 30-day mortality [11]. And second, in a pilot study in our animals, high doses of alcohol (2 g/kg) induced hemolysis and rapid death.

### Surgical preparation

Animal preparation and surgical procedures were the same as previously reported [18]. Briefly, the animals were premedicated with i.m. midazolam (0.25 mg/kg, Dormicum; Roche SA, Brussels, Belgium) and ketamine hydrochloride (20 mg/kg, Imalgine; Merial, Lyon, France). We then placed a peripheral venous line (14G, Surflo IV Catheter, Terumo Medical Company, Belgium) in the cephalic vein and inserted an oral endotracheal tube (I.D. 8 mm, Hi-Contour, Mallinckrodt Medical, Athlone, Ireland). Mechanical ventilation was provided (Servo 300 ventilator, Siemens-Elema, Solna, Sweden) using a tidal volume of 10 mL/kg, a respiratory rate of 15–25/min, a F_I_O_2_ of 0.25–0.80 titrated to keep PaCO_2_ between 35 and 45 mmHg and a positive end-expiratory pressure (PEEP) of 5 cmH_2_O. A heat-moisture exchanger (HMEF 750/S GE Healthcare, Helsinki, Finland) was used. The animals were anesthetized with midazolam (0.6–3.0 mg/kg/h), ketamine hydrochloride (8–40 mg/kg/h) and morphine (0.4–2.0 mg/kg/h) throughout the experiment. A neuromuscular blocker was also given (rocuronium, 10 μg/kg/h).

Under sterile conditions, we inserted an arterial catheter (Leader-Cath, 4.5 Fr, Vygon, Ecouen, France) in the right carotid artery and an introducer (Intro-Flex, 8.5 Fr, Edwards Lifesciences, Irvine, CA) in the right jugular vein for a 7 Fr pulmonary artery catheter (Swan-Ganz catheter, Edwards Lifesciences). Feces were collected via surgical perforation of the cecum. The cecum was then closed and a plastic tube (Beldico, Aye, Belgium) placed in the abdominal cavity for later introduction of feces. A flow probe (4PS, Transonic Systems, Ithaca, NY) was placed around the left renal artery through a lateral oblique incision. Two holes were drilled in the skull (1.2 mm, within 1 cm from the ear line at 1 cm lateral of midline) (Guangzhou Hekang Medical Instrument, Guangzhou, China) to insert a laser Doppler flowmetry probe (Oxyflo, Single Fiber Driver Probe, Optronix, London, England) and a microdialysis membrane catheter (CMA 20 Microdialysis Probe, CMA, Solna, Sweden).

### Sepsis induction and management

Baseline measurements of all variables were taken and autologous feces (1.5 g/kg) were then introduced into the abdominal cavity to induce severe peritonitis.

Fluid resuscitation consisting of 4–20 mL/kg/h of Ringer’s lactate solution (Hartmann, Baxter) and 6% hydroxyethyl starch (Voluven, Fresenius Kabi, Bad Homburg, Germany) in a 1:1 ratio was given to keep pulmonary artery balloon-occluded pressure at the baseline level. No vasopressors were given. Sedatives and analgesics (0.6 mg/kg/h midazolam, 8 mg/kg/h ketamine and 0.4 mg/kg/h morphine) were administered continuously. Hypoglycemia (blood glucose < 50 mg/dL) and hypokalemia (defined as potassium < 3.5 mmol/L) were corrected. Metabolic acidosis was defined as pH < 7.35 with bicarbonate < 24 mmol/L. Hyperchloremia was defined as a chloride concentration exceeding 107 mmol/L. Renal dysfunction was defined as a decrease in renal blood flow to < 75% of baseline and a urine output < 0.5 mL/kg.

### Measurements

Observations were continued until spontaneous death. MAP, mean pulmonary arterial pressure (MPAP) (displayed on a SC9000, Siemens, Munich, Germany), blood temperature and cardiac output (measured on Vigilance, Edwards Lifesciences, Irvine, CA) were recorded hourly. For cardiac output measurement, continuous cardiac output was measured automatically when core body temperature was < 41 °C, and using a bolus infusion (10 mL 4 °C of 0.9% saline) when the blood temperature increased > 41 °C. Derived variables were calculated using standard formulas. Arterial blood gas and lactate concentrations (Cobas b 123, Roche, Rotkreuz, Switzerland) were measured hourly. Every 4 h, citrated serum was separated by centrifugation (3000 rpm, 6 min) and stored at − 20 °C to measure coagulation variables in the biochemical laboratory of Erasme Hospital. Absolute values of renal blood flow (TS420, Transonic Systems) and laser Doppler flowmetry for brain regional microcirculation (Oxford Optronix OxyFlow 2000) were recorded hourly and normalized to baseline values. For microdialysis analysis, an artificial solution (Perfusion fluid CNS, CMA Microdialysis AB) was continually perfused at the rate of 0.3 μg/min (CMA402 Syringe Pump, CMA Microdialysis AB). One-hour effluent samples were immediately analyzed for glucose, lactate and pyruvate concentrations (ISCUS flex Microdialysis Analyzer, CMA Microdialysis AB). A microdialysis lactate/pyruvate ratio > 25 was used to define tissue ischemia in the brain cortex [19].

### Statistical analysis

In this exploratory trial, we did not perform an a priori calculation of the sample size, but selected the number of animals based on our previous experience with this animal model [18, 19]. Statistical analysis was performed using JMP® (Ver. 10. SAS Institute, Cary, NC). Continuous variables are presented as means ± standard deviation (SD) or median [25, 75% interquartile range (IQR)].

To estimate the influence of ethanol administration over the observational period, a mixed-effects polynomial regression model with restricted maximum likelihood (REML) estimation was used to examine the mean differences in time-series variables between the groups. The effects of time and group, as well as the interaction between group and time, were tested as fixed effects and subjects were introduced as random effects. If there were significant differences, a Student’s t-test (two-tailed) was used for comparison of the mean of these variables at each point between the two groups. Time to develop several predefined organ failure parameters and survival time between groups was tested using a log-rank test. A *p* value of < 0.05 was considered statistically significant.

## Results

The ethanol and control groups had similar characteristics at the start of ethanol administration and at the moment of feces injection (Table 1 and in Additional file 1: Table S1). In the ethanol group, the ethanol concentration in blood was largest at the time of feces injection (137 ± 29 mg/dL) and ethanol was no longer detectable at 12 h (Additional file 1: Figure S1).

**Table 1:** Evolution of measured variables in the two groups (ethanol, *n* = 7 vs. control, *n* = 7)

	Groups	-2 h	0 h (baseline = sepsis induction)	4 h	8 h	12 h	16 h	Mean diff. (p)^b^	
								Group	Group time interaction
Hemodynamics									
Heart rate (/min)	Ethanol Control	91 ± 16 88 ± 8	101 ± 21 100 ± 7	138 ± 28 130 ± 25	138 ± 19 122 ± 13	148 ± 23 122 ± 17	147 ± 28 149 ± 41	0.90	0.96
Mean arterial pressure (mmHg)	Ethanol Control	90 ± 7 93 ± 19	92 ± 12 96 ± 7	87 ± 11 100 ± 16	63 ± 16 92 ± 18	52 ± 12 69 ± 17	47 ± 11 52 ± 15	0.60	0.11
Pulmonary artery occlusion pressure (mmHg)	Ethanol Control	5 ± 4 6 ± 2	4 ± 2 4 ± 2	2 ± 1 4 ± 2	3 ± 2 5 ± 3	3 ± 2 5 ± 3	5 ± 5 7 ± 2	0.33	0.34
Cardiac index (L/min/m^2^)	Ethanol Control	4.5 ± 1.0 4.4 ± 0.7	5.8 ± 1.6 4.9 ± 1.2	5.7 ± 1.2 5.6 ± 1.0	6.2 ± 1.6 6.3 ± 1.0	5.6 ± 1.9 5.6 ± 1.7	6.3 ± 3.1 4.5 ± 2.0	0.82	0.90
Systemic vascular resistance index (dyn·s/cm^5^·m^2^)	Ethanol Control	2414 ± 737 2665 ± 382	2929 ± 738 2948 ± 632	1845 ± 292 2096 ± 757	1530 ± 355 1790 ± 655	1280 ± 336 1369 ± 316	991 ± 249 1567 ± 631	0.33	0.92
Other parameters									
Blood temperature (°C)	Ethanol Control	39.0 ± 0.7 38.4 ± 0.7	39.3 ± 1.0 38.9 ± 1.1	39.9 ± 1.1 39.5 ± 1.1	40.5 ± 1.2 40.0 ± 1.2	41.0 ± 1.0 40.6 ± 1.1	41.0 ± 1.3 40.9 ± 1.1	0.31	0.02
Mixed venous oxygen saturation (%)	Ethanol Control	69.2 ± 9.7 66.4 ± 9.2	70.7 ± 8.6 69.5 ± 8.9	74.3 ± 7.6 74.8 ± 4.8	71.0 ± 15.6 76.5 ± 6.4	62.0 ± 20.2 72.7 ± 8.6	63.9 ± 13.6 60.5 ± 16.8	0.63	0.45
Cumulative fluid administration (mL/kg)	Ethanol Control	– –	0 0	41 ± 7 38 ± 6	96 ± 8 90 ± 13	144 ± 20 144 ± 22	207 ± 32 176 ± 27	1.00	0.01
Urine output (mL/kg/h)	Ethanol Control	– –	1.9 ± 1.5 2.0 ± 1.3	1.7 ± 1.0 1.6 ± 0.8	1.1 ± 1.0 1.6 ± 1.2	0.1 ± 0.2 0.7 ± 0.4	0.1 ± 0.1 0.3 ± 0.5	0.90	0.73
PaO_2_/F_I_O_2_ ratio	Ethanol Control	447 ± 54 453 ± 80	433 ± 71 441 ± 67	393 ± 81 409 ± 43	305 ± 115 389 ± 62	235 ± 121 344 ± 76	216 ± 118 261 ± 126	0.66	0.12
Arterial pH	Ethanol Control	7.42 ± 0.07 7.44 ± 0.05	7.40 ± 0.08 7.43 ± 0.07	7.33 ± 0.09 7.43 ± 0.07	7.29 ± 0.09 7.40 ± 0.07	7.17 ± 0.13 7.33 ± 0.09	7.09 ± 0.15 7.17 ± 0.20	0.93	0.08
Arterial lactate (mmol/L)	Ethanol Control	0.6 ± 0.4 0.6 ± 0.3	1.5 ± 0.9 0.8 ± 0.6	3.5 ± 1.7 1.0 ± 0.8	4.0 ± 2.6 1.0 ± 0.7	6.1 ± 5.0 2.2 ± 1.7	5.6 ± 4.0 5.6 ± 3.5	0.93	0.09
Organ/tissue flow									
Renal artery flow ^a^	Ethanol Control	0.90 ± 0.17 0.94 ± 0.16	1 1	1.04 ± 0.27 1.14 ± 0.29	0.83 ± 0.60 1.24 ± 0.42	0.46 ± 0.32 0.83 ± 0.33	0.36 ± 0.09 0.43 ± 0.34	0.96	0.19
Brain laser-Doppler flowmetry ^a^	Ethanol Control	1.02 ± 0.17 1.08 ± 0.20	1 1	1.17 ± 0.11 1.14 ± 0.14	1.10 ± 0.32 1.08 ± 0.19	1.09 ± 0.34 0.93 ± 0.28	0.71 ± 0.10 0.77 ± 0.55	0.46	1.00
Cerebral microdialysis analysis									
Glucose (mg/dL)	Ethanol Control	– –	30.7 ± 13.2 19.8 ± 11.6	34.6 ± 11.2 23.8 ± 12.5	26.6 ± 9.0 19.9 ± 11.3	19.6 ± 7.7 19.6 ± 16.5	16.6 ± 4.5 17.4 ± 23.5	0.17	0.12
Lactate (mmol/L)	Ethanol Control	– –	2.1 ± 0.9 1.4 ± 0.8	3.8 ± 1.3 3.4 ± 0.9	4.5 ± 1.6 4.3 ± 1.3	5.2 ± 2.2 4.6 ± 1.5	4.2 ± 2.1 6.1 ± 2.5	0.41	0.92
Pyruvate (μmol/L)	Ethanol Control	– –	136 ± 68 93 ± 39	234 ± 97 199 ± 107	287 ± 139 201 ± 119	259 ± 122 185 ± 83	136 ± 72 125 ± 83	0.36	0.88
Lactate/pyruvate ratio	Ethanol Control	– –	16 ± 5 19 ± 10	17 ± 5 21 ± 9	17 ± 6 29 ± 18	21 ± 7 31 ± 17	32 ± 10 55 ± 35	0.51	0.79

Values are presented as mean ± SD. ^a^, relative flow with baseline flow defined as 1. ^b^, mixed model

### General course

The two groups had similar cardiac filling pressures, fluid balance and cardiac index throughout the study period (Table 1 and Additional file 1: Table S2). MAP reached < 65 mmHg earlier in the ethanol group (Table 2).

**Table 2:** Time to develop predefined major abnormalities

	Ethanol, *n* = 7	Control, *n* = 7	p (Log-rank)
MAP < 65 mmHg	8 [7–12]	14 [11–20]	0.03
SVRI < 1200 dyn·s/cm^5^·m^2^	11 [9–13]	14 [10–16]	0.19
Renal blood flow < × 0.75^a^	9 [6–13]	13 [12–15]	0.05
Hourly UO < 0.5 mL/kg	10 [7–12]	13 [12–13]	0.01
pH < 7.25	8 [4–15]	15 [13–20]	0.05
BT increase > + 1.5 °C^a^	10 [8–16]	11 [7–12]	0.54
PaO_2_/F_I_O_2_ ratio < 200	13 [11–15]	17 [13–20]	0.13
Brain LD < × 0.8^a^	16 [13–16]	16 [13–19]	0.49
Brain MD LPR > 25	14 [10–16]	12 [8–14]	0.59
Death	16 [14–22]	20 [19–24]	0.11

All data (hours) are presented as median [IQR]. ^a^, compared with baseline (defined as the time of feces injection) values

*BT* blood temperature, *LD* laser Doppler, *LPR* lactate pyruvate ratio, *MAP* mean arterial pressure, *MD* microdialysis, *SVRI* systemic vascular resistance index, *UO* urine output

**Fig. 1:**
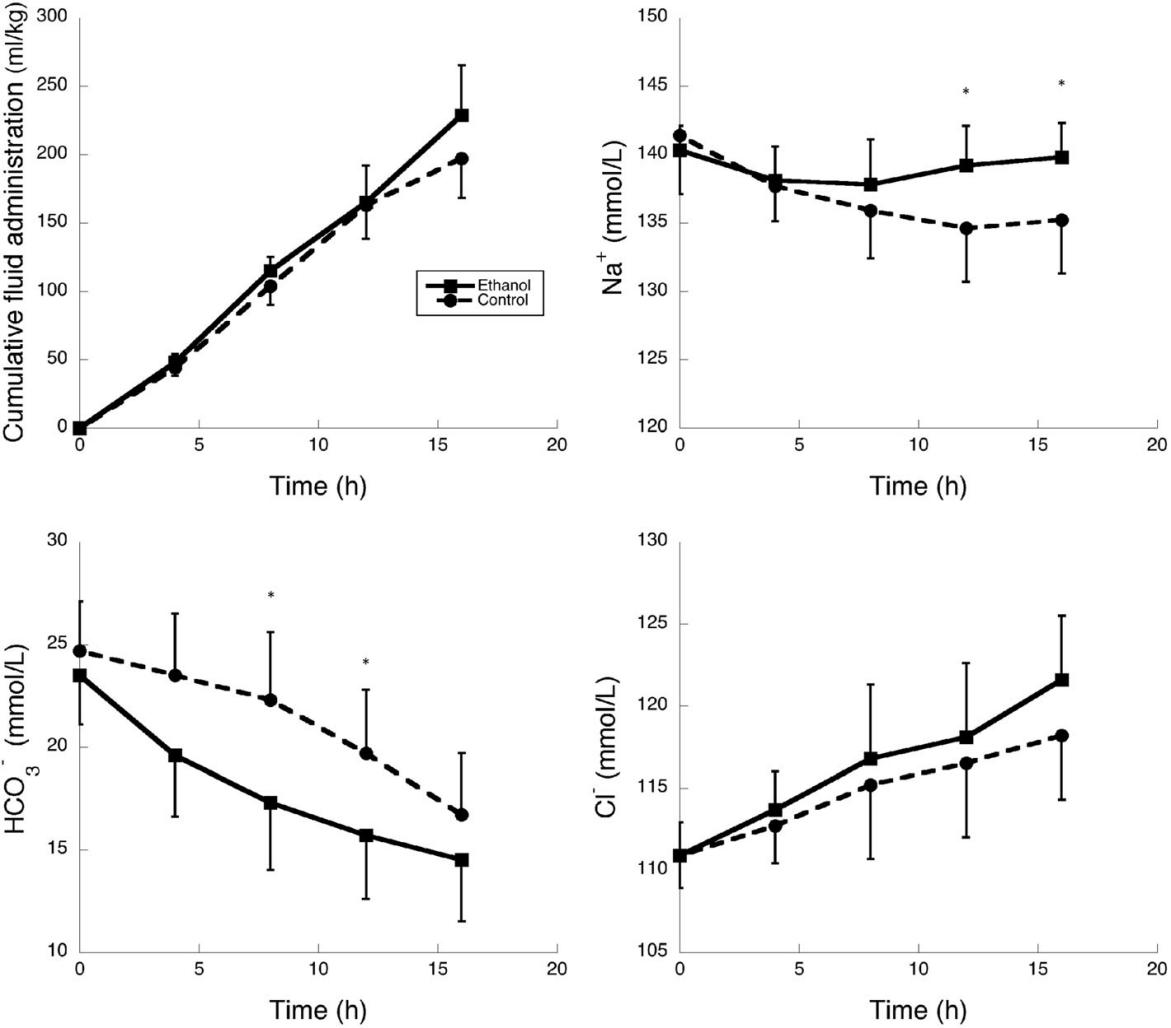
Cumulative fluid administration, arterial sodium, bicarbonate and chloride concentrations in the two groups. * difference between groups at the *p* < 0.05 level

Metabolic acidosis developed earlier in the ethanol than in the control group (Tables 1 and 2, Fig. 1), and was associated with a much earlier increase in arterial lactate concentrations in the ethanol group (from 1 h after feces injection) than in the control group (from 12 h after feces injection) (Table 1). The anion gap (Additional file 1: Table S2) and arterial sodium concentration (Fig. 1) decreased less in the ethanol group than in the control group. Hyperchloremia developed in both groups (Fig. 1).

### Renal function

Renal blood flow decreased to < 75% of baseline (9 [6–13] vs 13 [12–15] h, *p* = 0.05) and urinary output to < 0.5 mL/kg (10 [7–12] vs 13 [12–13] h, *p* = 0.01) earlier in the ethanol than in the control group (Table 2).

### Brain regional perfusion and lactate/pyruvate

Brain perfusion assessed by laser Doppler flowmetry was similar in the two groups throughout the study (Table 1). Brain microdialysis analysis showed that interstitial glucose decreased and lactate increased in both groups. Interstitial pyruvate increased initially in both groups and then decreased from about 12 h; as a result, the lactate/pyruvate ratio started to increase at the end of the observation period. All these changes were similar in the two groups (Table 1).

### Survival time

The survival time was shorter in the ethanol group than in the control group (16 vs. 20 h), but the differences were not statistically significant (*p* = 0.11) (Table 2).

## Discussion

In this ovine sepsis model, we showed that acute ethanol intoxication induced earlier development of hypotension and lactic acidosis, and earlier decrease in bicarbonate and pH. There was also an earlier decrease in renal blood flow and urine output in the animals who received ethanol; however, ethanol did not affect brain cortex perfusion or lactate/pyruvate.

Acute ethanol intoxication induced earlier hypotension despite adequate fluid resuscitation in the two groups. This effect may be explained by ethanol-induced upregulation of inducible nitric oxide synthase (iNOS). In a rat endotoxemia model, ethanol increased myocardial and aortic NOS, aggravating hypotension and decreasing cardiac output [20, 21]. Conversely, also in rats, Ajisaka et al. [8] using low dose ethanol (100 mg/kg/h for 2 h) and Greenberg et al. [9] using high dose ethanol (2.2–5.5 g/kg, intraperitoneally) both showed attenuation of endotoxin-induced hypotension, suggesting ethanol-induced suppression of endothelial NOS expression. In addition to obvious differences, such as different species and sepsis models, another possible reason for the conflicting results may be related to management of fluid status: for example the rat endotoxemia model used by Greenberg et al. [9] had inadequate fluid resuscitation and hemodynamic monitoring, which could potentially weaken the interpretation of their results. Furthermore, oxidative stress and autonomic dysregulation were shown to be responsible for the hypotension observed in a rat model of acute ethanol exposure (1 g/kg, intragastric) [22]. Impaired release of arginine vasopressin, which may be the result of augmented central NO inhibition, may also play a role [23]. These possible mechanisms need to be confirmed in future studies.

In addition to earlier hypotension, acutely intoxicated animals also showed an earlier onset of lactic acidosis. In a rat blunt (thoracic) chest trauma and hemorrhagic shock model, Wagner et al. showed that acute ethanol intoxication induced both respiratory and metabolic acidosis [24]. In guinea pigs with hemorrhagic shock, acute ethanol intoxication also exacerbated lactic acidosis [25, 26]. Zehtabchi et al. reported a higher prevalence of lactic acidosis in ethanol-intoxicated patients than in other patients in the emergency department [27]. Respiratory acidosis is related to depression in respiratory rate [28], whereas metabolic acidosis may be related to alcoholic ketoacidosis associated with an increased anion gap [29] and renal dysfunction [24], both of which were observed in our study. Because we used mechanical ventilation to keep PaCO_2_ within normal ranges, we cannot exclude the possibility that respiratory acidosis may have occurred in our model.

Acute ethanol intoxication resulted in the earlier development of renal dysfunction, suggested by an earlier decrease in renal blood flow and urine output. This was likely due to the earlier decrease in arterial pressure. However, brain cortex perfusion and metabolism and mortality were not affected, possibly because kidney autoregulation may not be as effective as cerebral autoregulation. Other possible explanations could be that the microcirculation in the brain was impaired less than that in the kidney. Finally, we cannot exclude the possibility that the brain may be able to use ethanol metabolites, such as ketone, more efficiently than the kidney.

The shorter survival time in the ethanol-intoxicated animals did not reach statistical significance possibly because the number of animals was too small, but we did not believe it was justified to add animals for this reason, especially since this observation is compatible with other reports. Woodman et al. showed that endotoxin shortened survival in ethanol intoxicated pigs [10]. Nishida et al. reported that acute ethanol administration increased 24-h lethality in a mouse sepsis model from 40 to 92% [30]. Possible mechanisms to explain the shortened survival could be related to the effects of acute ethanol exposure on innate and adaptive immunity [31–33] and oxidative stress [34, 35]. However, in a series of 11,850 medical and surgical ICU patients, Stehman et al. [11] reported that patients with acute ethanol intoxication had a lower risk of blood stream infection, sepsis and organ dysfunction, and death than the other patients, raising potential questions regarding the translation of these animal results to the clinical setting.

Ethanol metabolism is similar in sheep and humans [36]. Nevertheless, our study has several limitations. First, the extent of ethanol-induced immune changes may depend on the route of administration, the amount and duration of ethanol consumed, and the strain and sex of animal species used [37]. Second, oral intake of ethanol may also induce gut mucosal injury, which could further harm the host. Furthermore, depending on the administration route (oral, intra-peritoneal or intra-venous), different doses (from 10 mg/kg to 6 g/kg) have been used in acute ethanol intoxication models. We chose to give only one dose (1 g/kg) over two hours rather than multiple doses, which may limit interpretation of our results given the relatively mild level of ethanol intoxication. Third, in our anesthetized sheep model, we do not give antibiotics or surgical treatment for peritonitis in order to achieve a uniformly lethal outcome. Hence, we could not study the long-term effects of ethanol intoxication. Moreover, the use of previously healthy sheep may not translate readily to the complex ICU patient with comorbidities. Fourth, HES solutions have been associated with potential harmful renal effects in clinical studies [38]; however, some colloid is necessary in this model to limit edema formation and we are unable to given human albumin. Nevertheless, the amounts of HES administered were small and given over a short time period so we do not believe they will have had a major impact on renal function in this model. Finally, we cannot address all the possible mechanisms involved because we did not measure ethanol metabolites or mediators, including inflammatory factors.

## Conclusion

In a clinically relevant ovine model of sepsis, acute ethanol intoxication resulted in earlier development of shock, and was associated with reduced renal blood flow and urine output, but did not alter brain perfusion or metabolism.

## Additional file

Additional file 1: Supplementary Content including: **Table S1.** Animals at baseline. **Figure S1** Blood ethanol concentrations over time. **Table S2.** Evolution of other measured variables in the two groups. (DOCX 45 kb)

## Abbreviations

iNOS: Inducible nitric oxide synthase
MAP: Mean arterial pressure
MPAP: Mean pulmonary artery pressure
