# Supplementary material for: Effects of acute ethanol intoxication in an ovine peritonitis model
Source: BMC Anesthesiol. 2018 Jun 19;18:70. doi: 10.1186/s12871-018-0537-1 (PMC6009814; doi:10.1186/s12871-018-0537-1)
Supplement: Supplementary file 1 — Supplementary Content including: Table S1. Animals at baseline. Figure S1 Blood ethanol concentrations over time. Table S2. Evolution of other measured variables in the two groups. (DOCX 45 kb) [file 12871_2018_537_MOESM1_ESM.docx]

**Supplementary Content**

Effects of acute ethanol intoxication in an ovine peritonitis model

Hosokawa et al

**Table S1**. Animals at baseline

|  | Ethanol, n = 7 | Control, n = 7 |
| --- | --- | --- |
| Body weight, kg | 27 ± 5 | 29 ± 3 |
| Time until feces injection, hours$ | 3.5 ± 0.3 | 3.3 ± 0.4 |
| Fluid administration, mL$ | 711 ± 291 | 691 ± 270 |
| Urine, mL$ | 182 ± 112 | 227 ± 230 |

Data are presented as mean ± SD. $, From premedication until feces injection.

**Fig. S1.** Blood ethanol concentrations over time


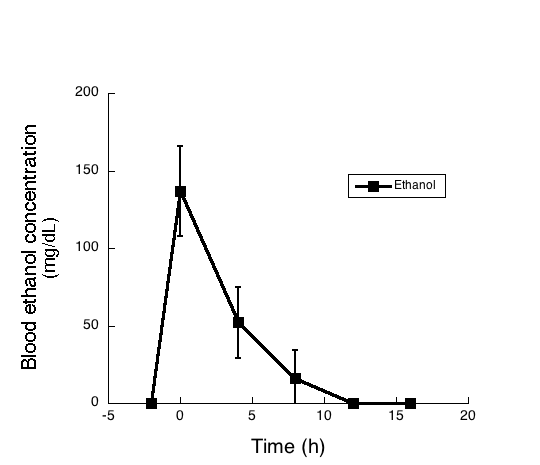


Table S2. Evolution of other measured variables in the two groups

|  |  | Groups | -2h | 0h (baseline) | 4h | 8h | 12h | 16h | Mean diff. (p)¶ | |
| --- | --- | --- | --- | --- | --- | --- | --- | --- | --- | --- |
|  |  |  |  |  |  |  |  |  | Group | Group time int. |
| Hemodynamics | |  |  |  |  |  |  |  |  |  |
|  | Mean pulmonary artery pressure (mmHg) | Ethanol  Control | 15 ± 2  15 ± 2 | 14 ± 2  14 ± 2 | 20 ± 4  18 ± 3 | 21 ± 3  21 ± 5 | 23 ± 3  22 ± 2 | 20 ± 2  25 ± 2 | 0.74 | 0.44 |
|  | Right atrial pressure (mm Hg) | Ethanol  Control | 1 ± 1  2 ± 2 | 0 ± 0  1 ± 2 | 0 ± 1  1 ± 1 | 1 ± 1  2 ± 2 | 2 ± 2  2 ± 2 | 5 ± 6  2 ± 3 | 0.71 | 0.84 |
|  | Stroke volume index (mL/beat/m^2^) | Ethanol  Control | 50 ± 8  50 ± 7 | 56 ± 9  49 ± 11 | 43 ± 13  44 ± 9 | 46 ± 15  52 ± 7 | 40 ± 16  46 ± 12 | 44 ± 25  30 ± 10 | 0.90 | 0.34 |
|  | Pulmonary vascular resistance index (dyn·s/cm^5^/m^2^) | Ethanol  Control | 300 ± 47  255 ± 33 | 295 ± 80  288 ± 122 | 359 ± 69  306 ± 74 | 415 ± 109  286 ± 95 | 520 ± 199  389 ± 195 | 551 ± 406  468 ± 135 | 0.41 | 0.14 |
|  | Oxygen delivery index (mL/min/m^2^) | Ethanol  Control | 557 ± 103  550 ± 140 | 693 ± 163  627 ± 199 | 797 ± 174  731 ± 128 | 838 ± 239  836 ± 152 | 757 ± 317  780 ± 199 | 796 ± 412  572 ± 215 | 0.81 | 0.95 |
|  | Oxygen consumption index (mL/min/m^2^) | Ethanol  Control | 185 ± 45  190 ± 17 | 206 ± 46  193 ± 42 | 214 ± 45  202 ± 54 | 208 ± 39  213 ± 55 | 205 ± 35  214 ± 53 | 204 ± 57  167 ± 45 | 0.62 | 0.49 |
|  | Oxygen extraction index (%) | Ethanol  Control | 30.3 ± 9.1  33.6 ± 9.2 | 28.6 ± 7.5  30.5 ± 8.9 | 24.8 ± 6.2  25.2 ± 4.8 | 25.9 ± 11.2  23.5 ± 6.3 | 29.7 ± 13.0  26.8 ± 8.1 | 28.6 ± 9.6  31.3 ± 8.7 | 0.56 | 0.79 |
| Other parameters | |  |  |  |  |  |  |  |  |  |
|  | Hemoglobin (g/dL) | Ethanol  Control | 8.8 ± 1.3  8.6 ± 1.1 | 8.7 ± 1.1  8.8 ± 1.1 | 9.9 ± 0.8  9.3 ± 1.4 | 10.0 ± 1.1  9.4 ± 0.7 | 10.9 ± 1.3  10.1 ± 1.2 | 10.1 ± 1.5  10.9 ± 1.6 | 0.90 | 0.26 |
|  | Thoracopulmonary compliance (mL/cmH_2_O) | Ethanol  Control | 26 ± 7  26 ± 6 | 23 ± 4  23 ± 5 | 18 ± 3  19 ± 3 | 15 ± 2  17 ± 3 | 13 ± 3  15 ± 3 | 11 ± 2  13 ± 5 | 0.47 | 0.72 |
|  | PaO_2_ (mmHg) | Ethanol  Control | 147.8 ± 29.9  138.2 ± 28.8 | 134.3 ± 29.4  139.6 ± 22.0 | 124.2 ± 17.5  128.1 ± 12.1 | 102.4 ± 27.7  121.6 ± 15.4 | 96.9 ± 41.9  110.5 ± 16.0 | 89.0 ± 42.4  94.7 ± 27.3 | 0.42 | 0.98 |
|  | PaCO_2_ (mmHg) | Ethanol  Control | 40.9 ± 5.4  39.5 ± 2.3 | 38.9 ± 4.3  37.4 ± 2.8 | 37.6 ± 3.4  36.3 ± 2.6 | 36.5 ± 2.2  36.2 ± 1.7 | 43.7 ± 8.3  37.7 ± 2.7 | 48.0 ± 7.8  39.5 ± 4.0 | 0.83 | 0.79 |
|  | Base deficit (mmol/L) | Ethanol  Control | -1.4 ± 2.5  -1.9 ± 3.7 | 1.1 ± 3.3  -0.4 ± 5.2 | 5.8 ± 4.2  0.7 ± 3.9* | 8.6 ± 4.5  2.1 ± 4.6* | 12.2 ± 5.1  5.8 ± 5.3* | 14.7 ± 5.4  11.5 ± 7.2 | 0.90 | <0.01 |
|  | Anion gap with K^+^ (mmol/L) | Ethanol  Control | 10.3 (2.7)  9.0 (2.8) | 9.8 (2.1)  9.9 (5.1) | 8.9 (2.6)  5.4 (2.1)* | 8.0 (3.9)  2.0 (2.6)# | 10.4 (5.2)  2.5 (2.1)# | 9.1 (3.8)  6.1 (4.2) | 0.58 | <0.01 |
|  | Blood glucose (mg/dL) | Ethanol  Control | 48.7 (9.3)  59.2 (11.3) | 47.1 (20.9)  61.3 (16.9) | 64.7 (22.8)  57.7 (13.8) | 45.9 (9.8)  44.9 (11.9) | 39.3 (14.1)  49.7 (6.9) | 35.8 (2.2)  51.7 (12.1) | 0.34 | 0.78 |
| Coagulation | |  |  |  |  |  |  |  |  |  |
|  | APTT (sec) | Ethanol  Control | 29.2 ± 5.0  32.7 ± 4.2 | 30.2 ± 5.3  30.4 ± 3.4 | 36.7 ± 8.2  35.5 ± 3.9 | 65.3 ± 38.0  50.3 ± 7.5 | 89.8 ± 42.2  66.6 ± 11.4 | 120.5 ± 26.7  108.0 ± 39.4 | 0.98 | 0.48 |
|  | PT (%) | Ethanol  Control | 58.9 ± 5.7  52.0 ± 8.5 | 54.0 ± 7.2  56.1 ± 7.9 | 35.7 ± 8.1  40.9 ± 6.7 | 20.3 ± 9.0  31.1 ± 5.0* | 12.4 ± 7.0  18.9 ± 7.6 | 7.0 ± 0.0  10.5 ± 5.9 | 0.65 | 0.01 |
|  | Fibrinogen (mg/dL) | Ethanol  Control | 220.0 ± 58.3  212.0 ± 42.3 | 206.3 ± 70.1  201.5 ± 41.6 | 99.0 ± 96.7  118.5 ± 23.3 | 48.0 ± 61.9  55.3 ± 60.8 | 14.9 ± 39.3  0.0 ± 0.0 | - | 0.57 | 0.76 |

Values are presented as mean ± SD. ¶, mixed model. *, p < 0.05, #, p < 0.01 compared to the control group.
